# A lack of association between hyperserotonemia and the increased frequency of serum anti-myelin basic protein auto-antibodies in autistic children

**DOI:** 10.1186/1742-2094-8-71

**Published:** 2011-06-22

**Authors:** Gehan Ahmed Mostafa, Laila Yousef AL-Ayadhi

**Affiliations:** 1 Autism Research and Treatment Center, AL-Amodi Autism Research Chair, Department of Physiology, Faculty of Medicine, King Saud University, Riyadh, Saudi Arabia; 2 Department of Pediatrics, Faculty of Medicine, Ain Shams University, Cairo, Egypt

**Keywords:** Anti-myelin-basic protein antibodies, autism, autoimmunity, hyperserotonemia, serotonin

## Abstract

**Background:**

One of the most consistent biological findings in autism is the elevated blood serotonin levels. Immune abnormalities, including autoimmunity with production of brain specific auto-antibodies, are also commonly observed in this disorder. Hyperserotonemia may be one of the contributing factors to autoimmunity in some patients with autism through the reduction of T-helper (Th) 1-type cytokines. We are the first to investigate the possible role of hyperserotonemia in the induction of autoimmunity, as indicated by serum anti-myelin-basic protein (anti-MBP) auto-antibodies, in autism.

**Methods:**

Serum levels of serotonin and anti-MBP auto-antibodies were measured, by ELISA, in 50 autistic patients, aged between 5 and 12 years, and 30 healthy-matched children.

**Results:**

Autistic children had significantly higher serum levels of serotonin and anti-MBP auto-antibodies than healthy children (P < 0.001 and P < 0.001, respectively). Increased serum levels of serotonin and anti-MBP auto-antibodies were found in 92% and 80%, respectively of autistic patients. Patients with severe autism had significantly higher serum serotonin levels than children with mild to moderate autism (P < 0.001). Serum serotonin levels had no significant correlations with serum levels of anti-MBP auto-antibodies in autistic patients (P = 0.39).

**Conclusions:**

Hyperserotonemia may not be one of the contributing factors to the increased frequency of serum anti-MBP auto-antibodies in some autistic children. These data should be treated with caution until further investigations are performed. However, inclusion of serum serotonin levels as a correlate may be useful in other future immune studies in autism to help unravel the long-standing mystery of hyperserotonemia and its possible role in the pathophysiology of this disorder.

## 1. Introduction

Autoimmunity to CNS may have a pathogenic role in autism [1]. This may be indicated by the presence of brain-specific auto-antibodies in some autistic children [2-8]. There is also an increase in the frequency of autoimmune disorders among autistic families [9-15].

Serotonin is formed by hydroxylation and decarboxylation of tryptophan. Serotonin is known to play a role in brain development prior to the time it assumes its role as a neurotransmitter. Disruption of serotonergic development can leave permanent alterations in brain function and behavior. This may be the case in autism [16,17]. It was suggested that autism, without a discernible cause, may be a genetic disorder of serotonin metabolism. The interest in assessing serotonergic function in autism stems from its role in perception and filtering of sensory signals, social attachment and facilitation of formation of synapses which is crucial to acquire learning and memory [18]. Blood serotonin might serve as analogue marker for serotonergic function [19].

Serotonin, being well known for its role in depression, has been shown to modulate immune responses. Serotonin may contribute to asthma pathogenesis through reduction of Th1-type cytokines [20]. In addition, hyperserotonemia may promote autoimmunity through reduction of Th1-type cytokines. This may result in an imbalance of T-helper (Th)1/Th2 subsets toward Th2, which are responsible for the allergic response and the production of antibodies. Hyperserotonemia may also promote autoimmunity through initiation of the delayed-type hypersensitivity responses, which has been proposed as a pathological mechanism leading to autism [21]. Accordingly, modifiers of the serotonin transmitter system such as compounds that affect the serotonin transporter, prejunctional serotonin receptors or postsynaptic serotonin receptors might represent a novel treatment of asthma and autoimmune disorders [22].

With this background, this study was conducted to investigate the relationship between serum levels of serotonin and anti-myelin-basic protein (anti-MBP) auto-antibodies, which are possible indicators of autoimmunity to CNS, in a group of autistic children.

## 2. Methods

### Study population

This case-control study was conduced on 50 children who had classic-onset autism. The patients were fulfilling the criteria for the diagnosis of autism according to the 4th edition of the Diagnostic and Statistical Manual of Mental Disorders [23].

The autistic group comprised 41 males and 9 females. They were recruited from the Autism Research and Treatment Center, Faculty of Medicine, King Saud University, Riyadh, Saudi Arabia. Their ages ranged between 5 and 12 years (mean ± SD = 8.22 ± 2.28 years).

#### Inclusion criteria

1-Patients who had no associated neurological diseases (such as cerebral palsy, tuberous sclerosis).

2-Patients who had no associated metabolic disorders (eg. Phenylketonuria) because these associated comorbidities with autism may influence the results of serum serotonin and anti-MBP levels.

3-Patients who were not receiving any medications.

The control group comprised 30 age-and sex-matched apparently healthy children. They included 25 males and 5 females. They were the healthy older siblings of the healthy children who attend the Well Baby Clinic, King Khalid University Hospital, Faculty of Medicine, King Saud University, Riyadh, Saudi Arabia for routine follow up of their growth parameters. The control children were not related to the children with autism, and demonstrated no clinical findings suggestive of immunological or neuropsychiatric disorders. Their ages ranged between 5 and 12 years (mean ± SD = 8.23 ± 2.36 years).

The local Ethical Committee of the Faculty of Medicine, King Saud University, Riyadh, Saudi Arabia, approved this study. In addition, an informed written consent of participation in the study was signed by the parents or the legal guardians of the studied subjects.

### Study measurements

#### Clinical evaluation of autistic patients

This was based on the clinical history taking from the caregivers, clinical examination and neuropsychiatric assessment. In addition, the degree of the severity of autism was assessed by using the Childhood Autism Rating Scale (CARS) [24] which rates the child on a scale from one to four in each of fifteen areas (relating to people; emotional response; imitation; body use; object use; listening response; fear or nervousness; verbal communication; non-verbal communication; activity level; level and consistency of intellectual response; adaptation to change; visual response; taste, smell and touch response and general impressions). According to the scale, children who have scored 30-36 have mild to moderate autism (n = 17), while those with scores ranging between 37 and 60 points have severe autism (n = 33).

#### Assessment of serum serotonin levels

Serum serotonin levels were assayed by using serotonin EIA kit (Biosource Europe S.A. rue de l'industrie 8 1400 Nivelles Belgium). Principally, the competitive serotonin EIA kit uses the microtiter plate format. Serotonin is bound to the solid phase of the microtiter plate. Acylated serotonin and solid phase bound serotonin compete for a fixed number of antiserum binding sites. When the system is in equilibrium, free antigen and free antigen-antiserum complexes are removed by washing. The antibody bound to the solid phase serotonin is detected by anti-rabbit peroxidase. The substrate TMB/peroxidase reaction is read at 450 nm with a filter wave length 620 nm. The corresponding serotonin concentrations are determined from the standard curve by matching their mean absorbance readings with the corresponding serotonin concentrations in ng/ml. To increase accuracy, all samples were analyzed twice in two independent experiments to assess inter-assay variations and to ensure reproducibility of the observed results (P > 0.05).

#### Measurement of serum anti-myelin basic protein (anti-MBP) antibodies

This was done by using ELISA kit that allows for the specific measurement of human anti-MBP (Diagnostic Systems, Texas, USA). This assay recognizes recombinant and natural human MBP antibodies. Principally, the microtiter plate provided in this kit has been pre-coated with MBP protein. Samples are added to the appropriate microtiter plate wells and incubated. Horseradish Peroxidase (HRP) conjugated to anti-antibody is added to each microplate well and incubated. Finally a TMB (3,3'5,5' tetramethyl-benzidine) substrate solution is added to each well. Only those wells that contain anti-MBP and enzyme-conjugated anti-antibody exhibit a change in color. The enzyme-substrate reaction is terminated by the addition of a sulphuric acid solution and the color change is measured spectrophotometrically at a wave length of 450 nm ± 2 nm. To increase accuracy, all samples were analyzed twice in two independent experiments to assess inter-assay variations and to ensure reproducibility of the observed results (P > 0.05). No significant cross-reactivity or interference was observed.

### Statistical analysis

The results were analyzed by commercially available software package (Statview, Abacus concepts, inc., Berkley, CA, USA). The data were non-parametric, thus they were presented as median and interquartile range (IQR), which are between the 25^th ^and 75^th ^percentiles. Mann-Whitney test was used for comparison between these data. Chi-square test was used for comparison between qualitative variables of the studied groups. Spearman's rho correlation coefficient "r" was used to determine the relationship between different variables. For all tests, a probability (P) of less than 0.05 was considered significant. Patients were considered to have elevated serum serotonin or anti-MBP if their levels were above the highest cut-off values (84.05 ng/ml and 221.55 ng/ml, respectively) which were the 95^th ^percentiles of serum serotonin and anti-MBP levels of healthy controls as the distribution of the data was non-parametric.

## 3. Results

### Serum serotonin levels in autistic children and their relation to the degree of the severity of autism

**Figure 1:**
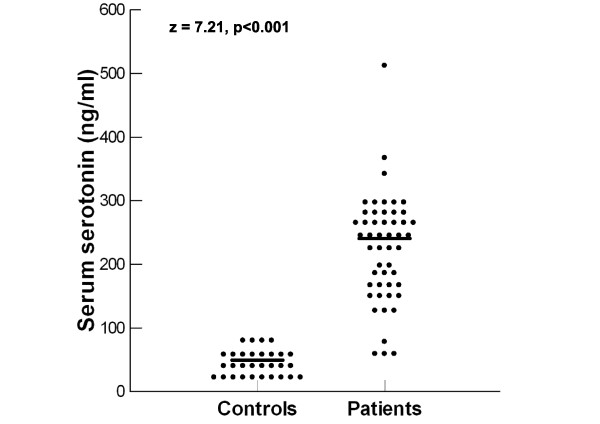
**Serum serotonin levels in autistic patients and healthy children**. Median value for each group is shown by a horizontal bar.

Autistic children had significantly higher serum serotonin levels [median (IQR) = 243.5 (119) ng/ml] than healthy controls [median (IQR) = 41 (31) ng/ml], P < 0.001 (Figure 1). Increased serum serotonin levels were found in 92% (46/50) of autistic patients.

**Figure 2:**
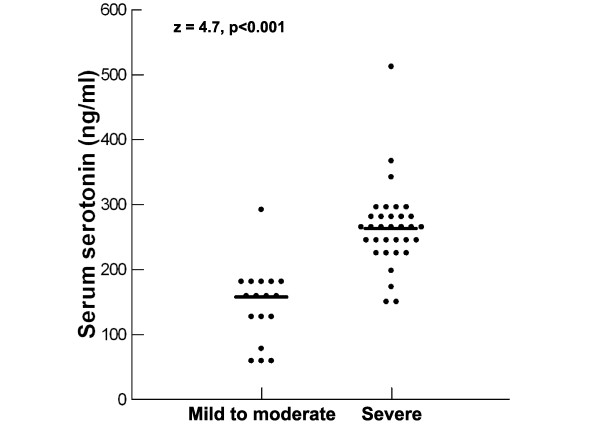
**Serum serotonin levels in relation to the degree of the severity of autism**. Median value for each group is shown by a horizontal bar.

Patients with severe autism had significantly higher serum serotonin levels [median (IQR) = 268 (46) ng/ml] than children with mild to moderate autism [median (IQR) = 160 (84) ng/ml], P < 0.001 (Figure 2). Also, the frequency of hyperserotonemia was significantly higher in children with severe autism (100%) than patients with mild to moderate autism (76.5%), P = 0.01.

Male and female autistic children had comparable values of serum serotonin [median (IQR) = 251 (115) ng/ml and 220 (92) ng/ml, respectively], P = 0.32. In addition, serum serotonin levels had no significant correlations with the age of the children with autism (P = 0.81).

### Serum levels of anti-MBP auto-antibodies in autistic children and its relation to the disease severity

Autistic children had significantly higher serum levels of anti-MBP auto-antibodies than healthy controls, P < 0.001 (table 1). According to the highest cut-off value of serum anti-MBP auto-antibodies, increased serum levels were found in 80% (40/50) of autistic patients.

**Table 1:** Serum levels of anti-MBP auto-antibodies in autistic patients and their relation to the degree of the severity of autism and hyperserotonemia.

	Serum anti-MBP (pg/ml) Median (IQR)	Z (P)
Healthy children (n = 30)	92 (49)	6.85
Autistic children (n = 50)	465.5 (348)	(< 0.001)
Patients with mild to moderate autism (n = 17)	334 (320)	1.45
Patients with severe autism (n = 33)	504 (328)	(0.15)
Patients with normal serum serotonin (n = 4)	382 (453.5)	0.36
Patients with hyperserotonemia (n = 46)	476.5 (348)	(0.74)

Anti-MBP, anti-myelin basic protein auto-antibodies. IQR, interquartile range.

There was a non-significant difference in serum levels of anti-MBP auto-antibodies between children with severe autism and patients with mild to moderate autism, P = 0.15 (table 1). In addition, there was a non-significant difference in the frequency of seropositivity of anti-MBP auto-antibodies between patients with severe autism (81.8%) and children with mild to moderate autism (76.5%), P = 0.46.

Male and female autistic children had comparable values of serum anti-MBP auto-antibodies [median (IQR) = 476 (346) ng/ml and 365 (361) ng/ml, respectively], P = 0.92. In addition, serum anti-MBP auto-antibodies had no significant correlations with the age of the children with autism (P = 0.75).

### Relationship between hyperserotonemia and the increased serum levels of anti-MBP auto-antibodies in autistic children

There was a non-significant difference in serum levels of anti-MBP auto-antibodies between autistic patients with hyperserotonemia and patients with normal serum serotonin levels, P = 0.74 (table 1). In addition, there was a non-significant difference in the frequency of seropositivity of anti-MBP auto-antibodies between autistic patients with hyperserotonemia (80.4%) and autistic patients with normal serum serotonin levels (75%), P = 0.6 (table 2). Furthermore, there were no significant correlations between serum levels of serotonin and anti-MBP in autistic patients (P = 0.39).

**Table 2:** Relationship between hyperserotonemia and the increased frequency of serum anti-MBP auto-antibodies in autistic children.

Patients with autism (n = 50)	Patients with normal serum serotonin (n = 4)	Patients with elevated serum serotonin (n = 46)	χ^2^ (P)
Patients with normal serum anti-MBP (n = 10)	1 (25%)	9 (19.6%)	0.07
Patients with elevated serum anti-MBP (n = 40)	3 (75%)	37 (80.4%)	(0.6)

Anti-MBP, anti-myelin basic protein antibodies.

## 4. Discussion

Autism without a discernible cause may be a genetic disorder of serotonin metabolism [18]. Immune abnormalities are also commonly observed in this disorder [1,25].

Elevated serotonin level in platelets, whole blood and serum is the most consistent biochemical abnormality found in autism [26]. In our series, autistic children had significantly higher serum serotonin levels than healthy controls (P < 0.001). In addition, increased serum serotonin levels were found in 92% (46/50) of autistic patients. Previous studies reported hyperserotonemia and increased platelet serotonin in some autistic children [27,28]. In one study hyperserotonemia was reported in 55% of a group of 80 autistic children aged between 3-12 years [29]. It was reported that 45% of the fathers, 51% of the mothers and 87% of the normal siblings of the autistic patients had elevated serotonin levels [30]. Also, parents of autistic children, who themselves had elevated blood serotonin levels, recorded significantly higher scores of depression and obsessive compulsive disorders than parents with normal serotonin levels [31]. Therefore, serotonin may represent a marker for familial autism [32].

In the present work, all autistic patients were not receiving any medications, including the selective serotonin reuptake inhibitors. This may explain the increased frequency of autistic patients with hyperserotonemia in this study. In addition, it was reported that serum serotonin levels are affected by age as postpubertal subjects had lower serum serotonin levels than prepubertal subjects [33]. Thus, the variations of the age, sex and race of the autistic patients between different studies may be another explanations of the variations of the frequency of hyperserotonemia between these studies.

Platelet hyperserotonemia has been detected in 25-60% of autistic children. The significant increase of the level of serotonin mRNA in the platelets of some autistic patients could suggest serotonin system dysregulation in some patients with autism [34]. Research observing the mechanism of hyperserotonemia indicated that it might result from increase serotonin uptake by platelets or decreased serotonin receptor binding sites on platelets [31].

Among the potential environmental factors, hyperserotonemia during pregnancy and its effect on brain development could be playing a role in autism. Hyperserotonemia during fetal development may result in a dysfunction of the hypothalamo-pituitary axis, affecting the amygdala as well as pro-social hormone oxytocin regulation. Dysfunction of the amygdala and abnormal oxytocin levels may underlie many clinical features of autism [35]. Hyperserotonemia, is a common biomarker in autism. The integrin β3 receptor subunit gene is a quantitative trait locus for the whole blood serotonin levels. Recent work shows that integrin β3 interacts with the serotonin transporter (SERT) in both the platelets and the midbrain in autism. Furthermore, multiple studies have now reported gene-gene interaction between the integrin β3 and SERT genes in association with autism [36]. SERT has received considerable attention as a potential risk locus for autism. One study reported a possible role of alphaIIbbeta3/SERT associations as well as alphaIIbbeta3 activation in control of SERT activity in vivo that may have broad implications for hyperserotonemia, cardiovascular disorders, and autism [37].

In the present work, patients with severe autism had significantly higher serum serotonin levels than patients with mild to moderate autism (P < 0.001). In addition, the frequency of hyperserotonemia was significantly higher in children with severe autism (100%) than patients with mild to moderate autism (76.5%), P = 0.01. This may indicate that the extent of the elevation of serum serotonin levels was closely linked to the degree of the severity of autism. Previous research also reported a close association between serum serotonin levels and the severity of autism [29]. It is not easy to determine whether hyperserotonemia is a mere consequence of autism or has a pathogenic role in the disease.

Some studies reported that autistic patients with elevated blood serotonin levels had elevated serotonin transport into platelets [38]. However, the high serotonin levels in the platelets do not necessarily mean that this translates to high levels in the brain. In fact, there are reasons to think otherwise. First: levels of 5-hydroxyindole acetic acid, the end product of serotonin metabolism, have not been found to be elevated in cerebrospinal fluid of autistic children [39]. Second: serotonin reuptake inhibitors which increase brain serotonin levels resulted in improvement of behavioral disorders and language acquisition in 59% of autistic children. Also, the decrease in brain serotonin following acute tryptophan depletion resulted in worsening of sterotyped movements in autistic children [18]. Third: positron emission tomography neuroimaging using a serotonin precursor, revealed diminished serotonin synthesis in the left hemisphere in 5 out of 7 autistic children [40]. Fourth: it was suggested that autistic children may have an autoimmune disorder affecting brain serotonin receptors since 7 out of 13 autistic children had CSF antibodies against serotonin receptors [41]. So, reduced brain serotonin content, which is important for language production and sensory integration, may represent one mechanism underlying the pathophysiology of autism [40].

Autoimmunity to CNS may have a pathogenic role in autism [1]. This may be indicated by the presence of brain-specific auto-antibodies in some autistic children [2-8]. In our series, increased serum levels of anti-MBP auto-antibodies were found in 80% (40/50) of autistic patients. A previous study reported seropositivity of anti-MBP protein auto-antibodies in 58% of autistic children [2]. These antibodies have been observed to be associated to viral serology as measles and herpes virus 6 [4], chlamydia pneumoniae and streptococcal M protein [42]. Other brain auto-antibodies such as anti-neuron-axon filament protein, anti-glial fibrillary acidic protein and anti-caudate nucleus were also reported to be increased in patients with autism [3,5]. More recently, seropositivity of anti-myelin-associated glycoprotein, anti-neuronal and anti-ganglioside M1 antibodies were reported in 62.5%, 54.5% and 74%, respectively of autistic children [6-8]. Despite of the fact that the origins of autoimmunity and the induction of the production of brain auto-antibodies in autism are unknown, the major histocompatibility complex genes and their products (e.g., HLA-DRB1 and C4B null alleles) might be involved [14,43].

MBP is a protein believed to be important in the process of myelination of nerves in the central nervous system. Interest in MBP has centered on its role in demyelinating diseases, particularly multiple sclerosis (MS). Several studies have shown a role for antibodies against MBP in the pathogenesis of MS [44,45].

The reason behind the formation of some brain auto-antibodies in some patients with autism is not fully understood. It is speculated that autoimmune reaction to neurons might be trigged by cross-reacting antigens in the environment resulting in the release of neuronal antigens. These neuronal antigens may result in induction of autoimmune reactions through the activation of inflammatory cells in genetically susceptible individuals. The environmental antigens may include food allergies to certain peptides such as gliadin, cow's milk protein and soy [46] infectious agents [1], heavy metals such as mercury [47] and Heavea Brasiliensis proteins in natural rubber latex [48]. Cross-reacting antigens in the environment may increase adhesion molecules on brain endothelial cells. Pre-existing autoreactive T-cells transmigrate across the blood-brain barrier (BBB) and induce activation of local antigen-presenting cells with production of cytokines that may result in oligodendrocyte damage and demyelination. These events may result in the release of antigens from neurofilaments that enter the circulation and induce the formation of plasma cells which produce antibodies against neuron-specific antigens. These antibodies may cross the BBB and combine with brain tissue antigens forming immune complexes that further damage the neurological tissue. Immunotherapy should be initiated in autistic children when a clue of autoimmunity is evidenced by the presence of auto-antibodies to CNS [42].

In our series, there was a non-significant difference in serum levels of anti-MBP auto-antibodies between children with severe autism and patients with mild to moderate autism, P = 0.15. In addition, there was a non-significant difference in the frequency of seropositivity of anti-MBP auto-antibodies between patients with severe autism (81.8%) and children with mild to moderate autism (76.5%), P = 0.46. These findings may indicate that the induction of the production of brain auto-antibodies in some patients with autism may occur regardless the degree of the disease severity.

Previous research had also found an increased frequency of autoimmunity in families of autistic children [9-15]. This may be an outstanding feature among autistic patients that points to their autoimmune background; the target in their case being the developing brain. This implies that in some families, immune dysfunction, perhaps induced by certain environmental triggers, could express itself in the form of autism in one of its offsprings.

To date, a definitive relationship between autism and autoimmunity has not been fully established. On the basis of the preliminary results reported in this study, however, there seems to be a suggestive evidence in support of the induction of autoimmune reaction against brain in some patients with autism. Additional investigation designed to expand on these data is warranted. Therapy in patients who are seropositive for serum auto-antibodies is directed at reducing the antibody concentration, blocking the effector mechanisms and depleting the monoclonal B cells. The recent availability of a monoclonal antibody suppressing B-cell clones, which is not myelosuppressive and does not cause secondary malignancies, allows for early targeted intervention [49]. Preliminary results suggest that this new line of therapy is well tolerated and is promising in the treatment of some patients [50,51]. Thus, we suggest that further studies with a larger subject population should be conducted to investigate the possiblr role of this therapy in autistic patients who have increased serum levels of anti-MBP auto-antibodies.

Serotonin has been shown to modulate some immune responses and hyperserotonemia may explain some of the abnormal cellular immune responses seen in autism. Serotonin has an important role in initiation of delayed-type hypersensitivity responses [52], which are important in autoimmunity, as serotonin initiates the activation of local endothelial cells to recruit effector T cells, and activates serotonin receptors on these recruited cells [53]. Some researchers found a correlation between serum serotonin levels and the presence of certain major histocompatibility complex (MHC) genes (the extended haplotype B44-DR4 and the C4B null allele) [54] which had previously reported to be associated with autism and to play an important role in the development of autoimmunity [14,43].

It is possible that a common factor could account for both hyperserotonemia and immune abnormalities seen in autism. Such common factor may be the serotonin transporter (5-HTT) which transports serotonin into platelets. 5-HTT is also found on immune cells where it can influence immune function [55]. Some studies found an association between autism and 5-HTT promotor gene polymorphisms [32]. However, the meta-analysis failed to find a significant overall association between either of the 5-HTT polymorphisms examined and autism [56]. In addition, hyperserotonemia may promote autoimmunity through reduction of the production of Th1-type cytokines resulting in an imbalance of T-helper (Th)1/Th2 subsets toward Th2, which are responsible for the production of antibodies [21]. This imbalance was reported in some autistic patients [1].

In this work, we have tried to find a possible relation between the elevated serum levels of serotonin and anti-MBP auto-antibodies. There was a non-significant difference in serum levels of anti-MBP auto-antibodies between autistic patients with hyperserotonemia and autistic patients with normal serum serotonin levels, P = 0.74. In addition, there was a non-significant difference in the frequency of seropositivity of anti-MBP auto-antibodies between autistic patients with hyperserotonemia (80.4%) and autistic children with normal serum serotonin levels (75%), P = 0.6. Furthermore, there was a non-significant correlation between serum serotonin and anti-MBP levels in autistic patients (P = 0.39). We could not trace data in the literature concerning the relationship between hypersrotonemia and brain auto-antibodies in autistic patients to compare our results. We are the first to study such a relationship. This warrants further research to determine the possible link between hyperserotonemia and the elevated serum levels of brain auto-antibodies which are detected in a subgroup of autistic patients.

The results of this study may indicate that hyperserotonemia is not one of the contributing factors to the increased frequency of anti-MBP auto-antibodies in some autistic children. However, this initial report can not rule out the possible association between hyperserotonemia and immune abnormalities that had been reported in autism, but rather raises additional questions. So, these data should be treated with caution until further investigations are performed. Therefore, studies should be conducted to investigate the possible association between hyperserotonemia and immune abnormalities in autism.

## Conclusions

Hyperserotonemia may not be one of the contributing factors to the increased frequency of serum anti-MBP auto-antibodies in some autistic children. These data should be treated with caution until further investigations are performed. However, inclusion of serum serotonin levels as a correlate may be useful in other future immune studies in autism to help unravel the long-standing mystery of hyperserotonemia and its possible role in the pathophysiology of this disorder. In addition, the possible role of blood serotonin lowering drugs in amelioration of immune abnormalities in hyperserotonemic autistic children should also be studied.

## List of abbreviations

(BBB): blood brain barrier; (CARS): Childhood Autism Rating Scale; (CNS): central nervous system; (IQR): interquartile range; (MBP): myelin basic protein; (SERT): serotonin transporter; (Th): T helper cells; (5-HTT): serotonin transporter.

## Competing interests

The authors declare that they have no competing interests.

## Authors' contributions

Both authors designed, performed and wrote the research. In addition, both authors have read and approved the final manuscript.
